# Apoptotic effect and cell arrest of deoxyshikonin in human osteosarcoma cells through the p38 pathway

**DOI:** 10.1111/jcmm.17764

**Published:** 2023-05-08

**Authors:** Ming‐Chang Hsieh, Yi‐Hsien Hsieh, Chia‐Hsuan Chou, Jia‐Sin Yang, Peace Wun‐Ang Lu, Tzu‐Yu Huang, Shun‐Fa Yang, Ko‐Hsiu Lu

**Affiliations:** ^1^ School of Medical Laboratory and Biotechnology Chung Shan Medical University Taichung Taiwan; ^2^ Department of Clinical Laboratory Chung Shan Medical University Hospital Taichung Taiwan; ^3^ Institute of Medicine Chung Shan Medical University Taichung Taiwan; ^4^ Department of Medical Research Chung Shan Medical University Hospital Taichung Taiwan; ^5^ Morrison Academy Taichung Taichung Taiwan; ^6^ Department of Orthopedics Chung Shan Medical University Hospital Taichung Taiwan; ^7^ School of Medicine Chung Shan Medical University Taichung Taiwan

**Keywords:** apoptosis, deoxyshikonin, osteosarcomas, p38

## Abstract

Osteosarcoma is the most common primary bone cancer that affects adolescents with early metastatic potential and drastically reduces their long‐term survival rate if pulmonary metastases are detected at diagnosis. The natural naphthoquinol compound deoxyshikonin exhibits anticancer properties, so we hypothesized that it has an apoptotic effect on osteosarcoma U2OS and HOS cells and studied its mechanisms. After deoxyshikonin treatment, dose‐dependent decreases in cell viability, induction of cell apoptosis and arrest in the sub‐G1 phase of U2OS and HOS cells were observed. The increases in cleaved caspase 3 expression and the decreases in X‐chromosome‐linked IAP (XIAP) and cellular inhibitors of apoptosis 1 (cIAP‐1) expressions after deoxyshikonin treatment in the human apoptosis array were identified in HOS cells, and dose‐dependent expression changes of IAPs and cleaved caspase 3, 8 and 9 were verified by Western blotting in U2OS and HOS cells. Phosphorylation of extracellular signal‐regulated protein kinases (ERK)1/2, c‐Jun N‐terminal kinases (JNK)1/2 and p38 expressions in U2OS and HOS cells was also increased by deoxyshikonin in a dose‐dependent manner. Subsequently, cotreatment with inhibitors of ERK (U0126), JNK (JNK‐IN‐8) and p38 (SB203580) was performed to show that p38 signalling is responsible for deoxyshikonin‐induced apoptosis in U2OS and HOS cells, but not via the ERK and JNK pathways. These discoveries demonstrate that deoxyshikonin may be a possible chemotherapeutic candidate to induce cell arrest and apoptosis by activating extrinsic and intrinsic pathways through p38 for human osteosarcoma.

## 1 INTRODUCTION

Potent metastatic osteosarcoma, which is mainly due to the metaphysis of the distal femur and the proximal tibia, is the most common histological form of primary bone malignancy in the paediatric age group with a high incidence in adolescents and a second high incidence in older adulthood.^1^, ^2^, ^3^ Clinically, plain radiographs and magnetic resonance imaging (MRI) with or without computed tomography (CT) of the entire affected bone, positron emission tomography and/or bone scan, chest CT and MRI or CT of the metastatic sites of the skeletal bones should be routinely surveyed for early detection of metastases and reduction of cancer‐associated deaths during osteosarcoma diagnosis.^4^ According to the radiological staging of osteosarcoma, the combination of neoadjuvant chemotherapy and limb preservation surgery has increased long‐term survival rates to approximately 68%.^5^, ^6^, ^7^ However, early lung metastases are responsible for one of the most lethal paediatric malignancies,^8^ so novel agents that target particular intracellular signalling pathways related to anti‐osteosarcoma cells are in urgent need of development.

Apoptosis, a process of programmed cell death, is hallmarked by distinctive morphological and biochemical characteristics that regulate physiological growth and tissue homeostasis.^9^, ^10^ By intracellular signalling pathways such as mitogen‐activated protein kinase (MAPK)/extracellular signal‐regulated protein kinase (ERK), c‐Jun N‐terminal kinase (JNK), and p38, apoptosis is modulated to promote healthy cell division, turnover, immunity and accurate development and cell death.^11^, ^12^, ^13^ In addition to initiator caspases 8 and 9 and effector caspases 3 and 7, several modulators of apoptosis, such as X‐chromosome‐linked IAP (XIAP) and cellular inhibitors of apoptosis 1 and 2 (cIAP‐1 and 2), are involved in the apoptotic pathway.^14^, ^15^, ^16^ Therefore, understanding the stress‐inducible molecules that trigger apoptosis in response to chemotherapy for human osteosarcoma attracts researchers to develop molecular‐targeted therapy through extrinsic and/or intrinsic pathways. Currently, most anti‐osteosarcoma strategies in clinical therapy focus on inducing apoptosis of cancer cells, whether they metastasize or not.

Shikonin, a natural naphthoquinone compound, extracted from the root of the Chinese herb *Lithospermum erythrorhizon*, family Boraginaceae, induces osteosarcoma cell death by apoptosis or necroptosis.^17^, ^18^, ^19^, ^20^ Because poor bioavailability and drug resistance prevent its clinical application, a newly natural derivative of shikonin deoxyshikonin, a hydroxy‐1,4‐naphthoquinone (C_16_H_16_O_4_), has anticancer effects in various cancer cell lines, including melanoma, leukaemia, oral, lung and colon cancer.^21^, ^22^, ^23^, ^24^, ^25^ However, the anti‐osteosarcoma effects of deoxyshikonin have not been fully delineated; therefore, we tested the hypothesis that deoxyshikonin induces apoptosis in human osteosarcoma cells and further explored its underlying pathway.

## 2 MATERIALS AND METHODS

### 2.1 Materials

Dulbecco's modified Eagle medium (DMEM), Eagle's minimum essential medium (MEM) and foetal bovine serum (FBS) for cell culture were obtained from Gibco Life Technologies. Specific antibodies for ERK1/2, JNK1/2, phosphorylated (p)‐ERK1/2, p‐JNK1/2, caspases 8 and 9, cleaved caspases 3, 8, and 9, XIAP and cIAP‐1 and 2 were purchased from Cell Signalling Technology. Antibodies specific for p38 and p‐p38 were purchased from Santa Cruz Biotechnology. The Fluorescein Isothiocyanate‐labelled (FITC) Annexin V Apoptosis Detection Kit I and Human Apoptosis Array Kit were obtained from BD Biosciences and R&D Systems, respectively. Other chemicals used in this study were purchased from Sigma‐Aldrich. Deoxyshikonin, with ≥98% purity of HPLC grade, was purchased from ChemFaces and dissolved in dimethyl sulfoxide (DMSO; Sigma‐Aldrich).

### 2.2 Cell culture and deoxyshikonin treatment

U2OS cells (osteogenic sarcoma; human, 15‐year‐old female), which were obtained from the Food Industry Research and Development Institute (Hsinchu, Taiwan), were cultured in DMEM supplemented with 10% FBS, 1% penicillin/streptomycin and 5 mL of glutamine. HOS cells (human osteosarcoma; 13‐year‐old female), which were obtained from the Food Industry Research and Development Institute, were cultured in Eagle's MEM supplemented with 10% FBS, 1% penicillin/streptomycin, and 5 mL glutamine. Cells were cultured at 37°C in a humidified atmosphere of 5% CO_2_ in an incubator,^26^ and treated with the experimental concentration range (2.5, 5, 10 and 20 μM) of deoxyshikonin in subsequent experiments.

### 2.3 Microculture tetrazolium assay

To identify the effect of deoxyshikonin on cell viability, an microculture tetrazolium (MTT) (3‐(4,5‐dimethylthiazol‐2‐yl)‐2,5‐diphenyltetrazolium bromide) colorimetric assay was performed to examine its cytotoxicity in U2OS and HOS cells. We seeded 8.5 × 10^4^/well U2OS cells and 6.5 × 10^4^/well HOS cells in 24‐well plates for 16 h and detected the experimental concentration range of deoxyshikonin at 37°C for 24 h. After the exposure period, we used the MTT assay as previously described.^27^, ^28^

### 2.4 Annexin V‐FITC apoptosis staining assay

After treatment with the experimental concentration range of deoxyshikonin, about 8.5 × 10^5^ U2OS and 6.5 × 10^5^ HOS cells were cultured in a 6 cm plate for 24 h. Subsequently, U2OS and HOS cells were harvested by trypsinization together with floating nonviable cells. We used the FITC Annexin V Apoptosis Detection Kit I based on the manufacturer's protocols (BD Biosciences) to determine the apoptotic effect of deoxyshikonin on U2OS and HOS cells, and also performed flow cytometry to analyse their cell cycles.^29^ In combination with annexin V‐FITC apoptosis staining and PI staining were measured to differentiate apoptosis from necrosis.

### 2.5 Human apoptosis array

To explore the underlying mechanism of induced apoptosis, we used the Human Apoptosis Array Kit to examine protein lysates from cells treated with vehicle or 20 μM deoxyshikonin‐treated cells for 24 h. According to the manufacturer's protocols (R&D Systems), the kit was used to detect 35 human apoptosis‐related proteins simultaneously. The captured proteins were detected with biotinylated detection antibodies and deposited on the nitrocellulose membrane and visualized with chemiluminescent detection reagents.^30^

### 2.6 Preparation of cell lysates and Western blot analysis

To verify the results of the human apoptosis array analysis and to explore the underlying signalling pathways of the molecular mechanism, Western blot analysis was performed. As previously described, we seeded 8.5 × 10^5^ U2OS and 6.5 × 10^5^ HOS cells in 6‐cm dishes for 16 h and examined them in the experimental deoxyshikonin concentration range for 24 h, and prepared total cell lysates of U2OS and HOS cells.^31^, ^32^, ^33^ We performed Western blot analysis using specific primary antibodies against the initiator and effector caspases 8, 9 and 3, cleaved caspases 8, 9 and 3 and specific antibodies against the unphosphorylated or phosphorylated forms of the three corresponding MAPKs (ERK1/2, JNK1/2 and p38) to identify possible pathways. Then we incubated the blots with horseradish peroxidase goat anti‐rabbit or anti‐mouse IgG for 1 h and measured the intensity of each band using densitometry.

### 2.7 Statistical analysis

One‐way analysis of variance (anova) with post hoc Tukey test were performed for more than two groups with equal sample sizes per group, respectively. Repeating each experiment at least three times (*n* ≥ 3) was performed independently. If *p* values <0.05, it was considered statistically significant.

## 3 RESULTS

### 3.1 Cytotoxicity of deoxyshikonin in U2OS and HOS cells

**FIGURE 1:**
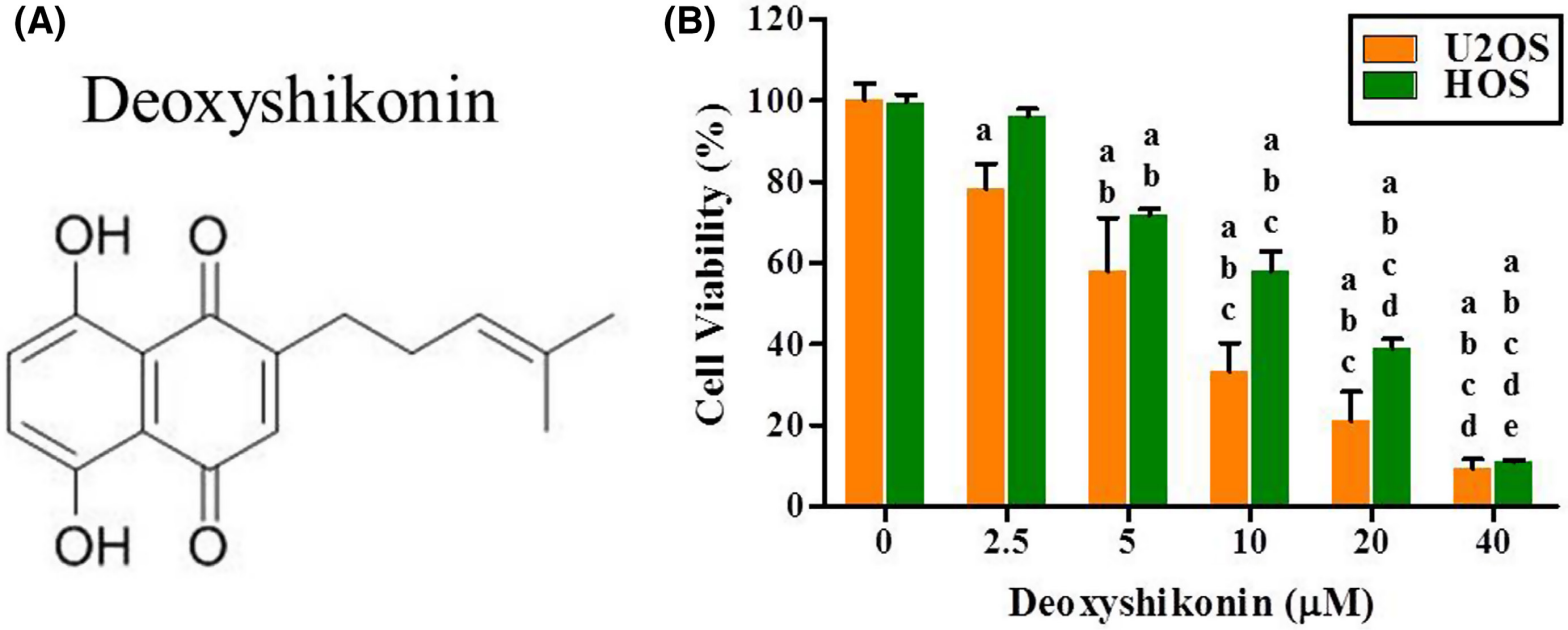
Analysis of deoxyshikonin on the cell viability in U2OS and HOS cells. The chemical structures of (A) deoxyshikonin were drawn. (B) The viability of U2OS and HOS cells treated with deoxyshikonin (0, 2.5, 5, 10, 20 and 40 μM) for 24 h was detected by MTT assay and the effects are illustrated after quantitative analysis. Results are shown as mean ± SD. anova analysis with Tukey posteriori comparison was used. U2OS (*n* = 4): *F* = 88.986, *p* < 0.001; HOS (*n* = 3): *F* = 515.677, *p* < 0.001. ^a^Significantly different, *p* < 0.05, when compared to control. ^b^Significantly different, *p* < 0.05, when compared to 2.5 μM. ^c^Significantly different, *p* < 0.05, when compared to 5 μM. ^d^Significantly different, *p* < 0.05, when compared to 10 μM. ^e^Significantly different, *p* < 0.05, when compared to 20 μM.

To assess deoxyshikonin (Figure 1A) cytotoxicity in osteosarcoma U2OS and HOS cells, we used the MTT assay. After 24 h of treatment, the viability of U2OS and HOS cells in the presence of concentrations of 2.5, 5, 10, 20 and 40 μM of deoxyshikonin was significantly different from those of controls (0 μM) (Figure 1B), and their relationships were dose‐dependent (*p* < 0.001 and *p* < 0.001). In 20 μM of deoxyshikonin, there were reductions of approximately 20% and 40% in U2OS and HOS cells, respectively, and approximately 90% in both cell lines in 40 μM of deoxyshikonin. Subsequently, we chose the experimental concentration range for deoxyshikonin in all subsequent experiments to explore its cytotoxic mechanisms.

### 3.2 Deoxyshikonin induces cell arrest in the sub‐G1 phase and apoptosis in U2OS and HOS cells

**FIGURE 2:**
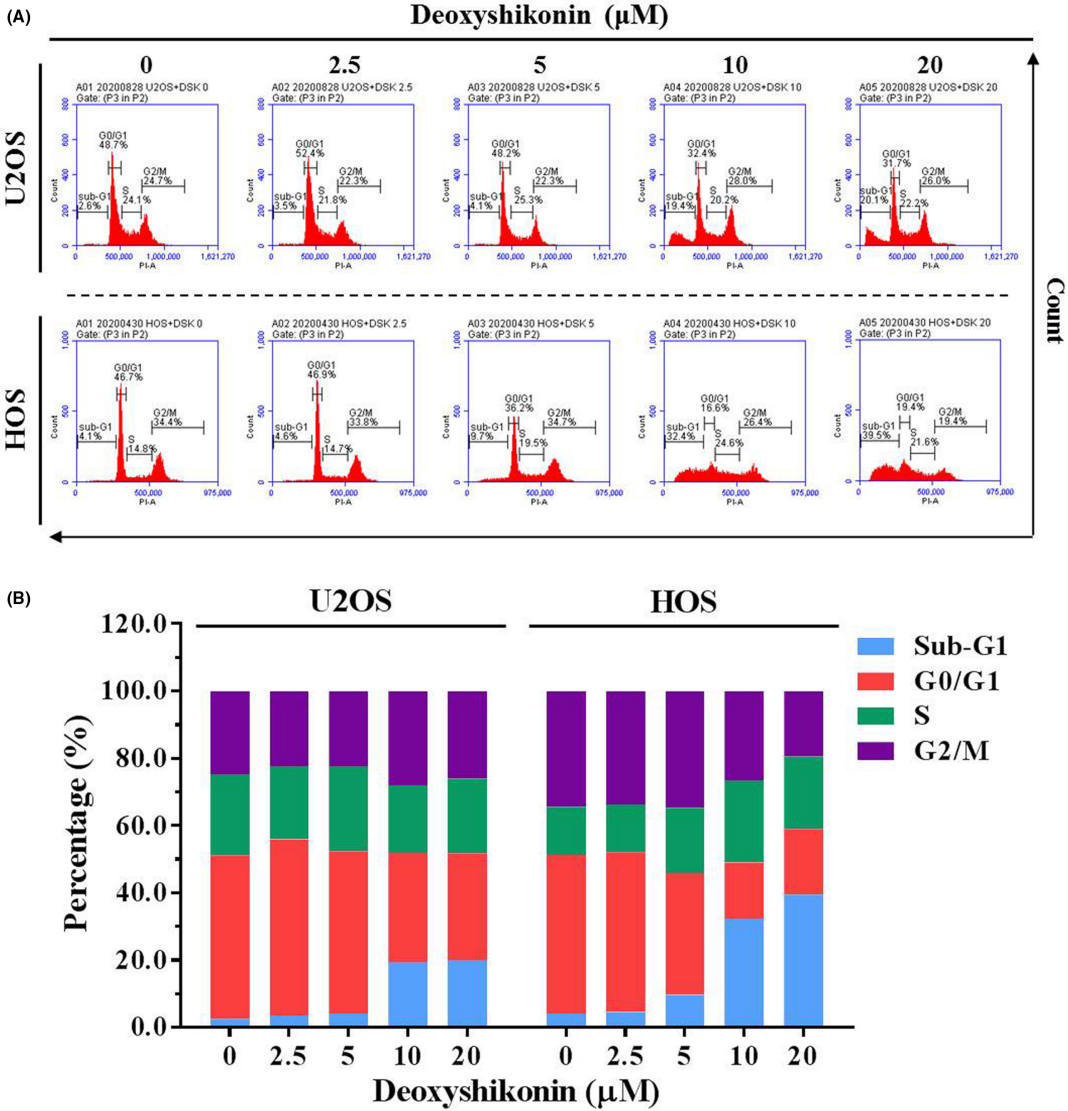
Analysis of deoxyshikonin on the cell cycle in U2OS and HOS cells. (A, B) U2OS and HOS were treated with deoxyshikonin (0, 2.5, 5, 10 and 20 μM) for 24 h and then subjected to flow cytometry for the cell cycle profile.

**FIGURE 3:**
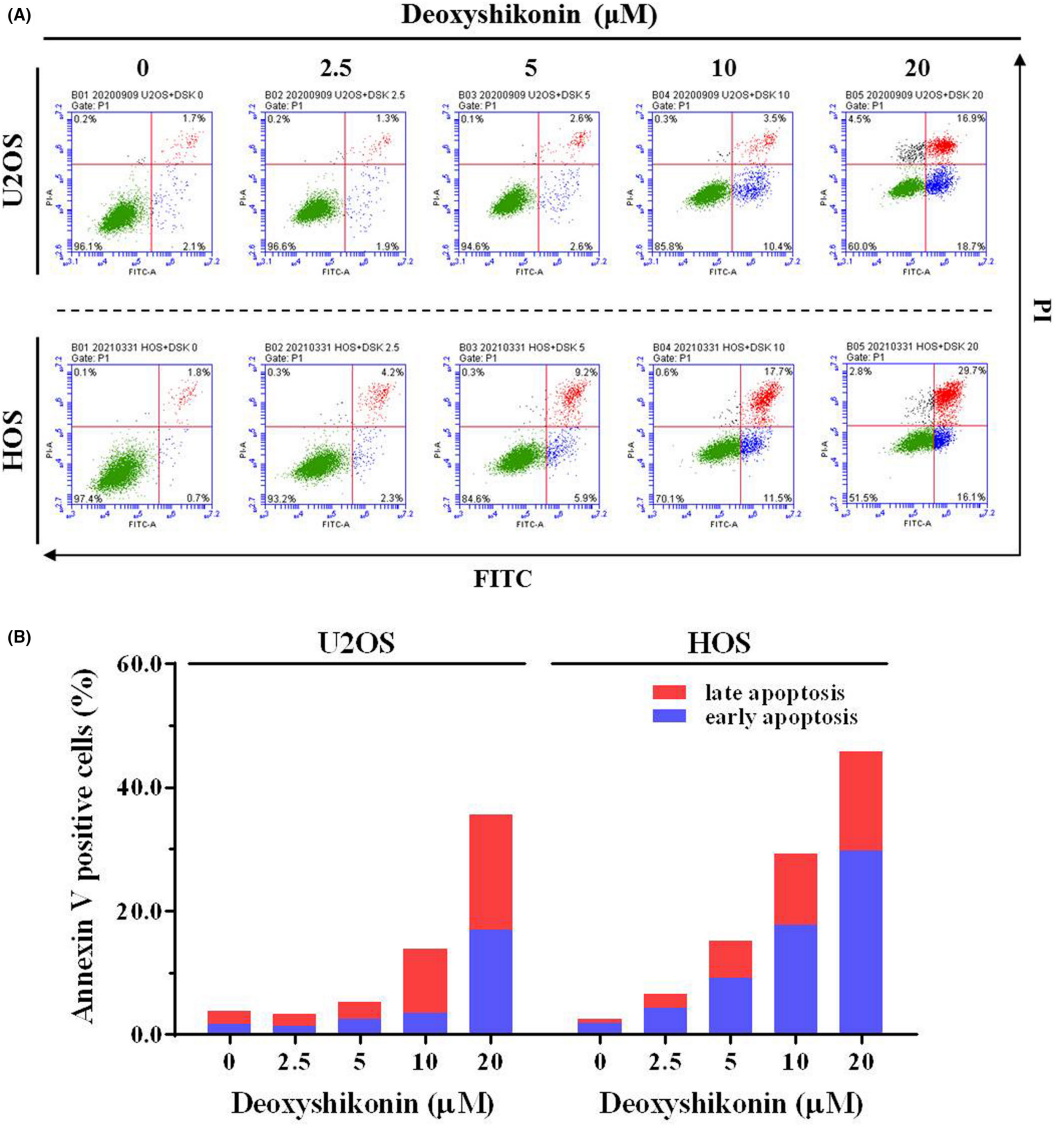
Analysis of deoxyshikonin on cell apoptosis in U2OS and HOS cells. (A) U2OS and HOS were treated with deoxyshikonin (0, 2.5, 5, 10 and 20 μM) for 24 h and then subjected to flow cytometry after PI and Annexin V‐FITC/PI staining to analyse DNA contents. The cell cycle profile of flow cytometry was subsequently quantified. Cells that were considered viable were FITC Annexin V and PI negative; cells that were in early apoptosis were FITC Annexin V positive and PI negative; and cells that were in late apoptosis or already dead were both FITC Annexin V and PI positive. (B) Thereupon quantitative analysis of early apoptosis and late apoptosis was summed up to differentiate apoptosis from necrosis.

To investigate unknown mechanisms of inhibition of deoxyshikonin on cell proliferation in U2OS and HOS, we used flow cytometry to examine the cell cycle. After PI staining, 20 μM of deoxyshikonin drastically increased the accumulation of the sub‐G1 phase cell cycle from 2.6% to 20.1% in U2OS cells and 4.1% to 39.5% in HOS cells, demonstrating that deoxyshikonin arrests the cell cycle in the sub‐G1 phase in U2OS and HOS cells (Figure 2). We also performed the annexin V‐FITC/PI apoptosis assay, which is crucial to differentiating different varieties of cell death, detecting apoptosis in earlier stages before gross morphological changes, and demonstrating the apoptotic effect of deoxyshikonin on human osteosarcoma cells. The results show that treatment with the experimental concentration range of deoxyshikonin for 24 h increases the percentage of apoptotic cells dose‐dependently (Figure 3). Using annexin V‐FITC/PI staining and flow cytometry, we confirmed that deoxyshikonin induces apoptosis in U2OS and HOS cells, and not necrosis.

### 3.3 Deoxyshikonin activates extrinsic and intrinsic apoptotic pathways in U2OS and HOS cells

**FIGURE 4:**
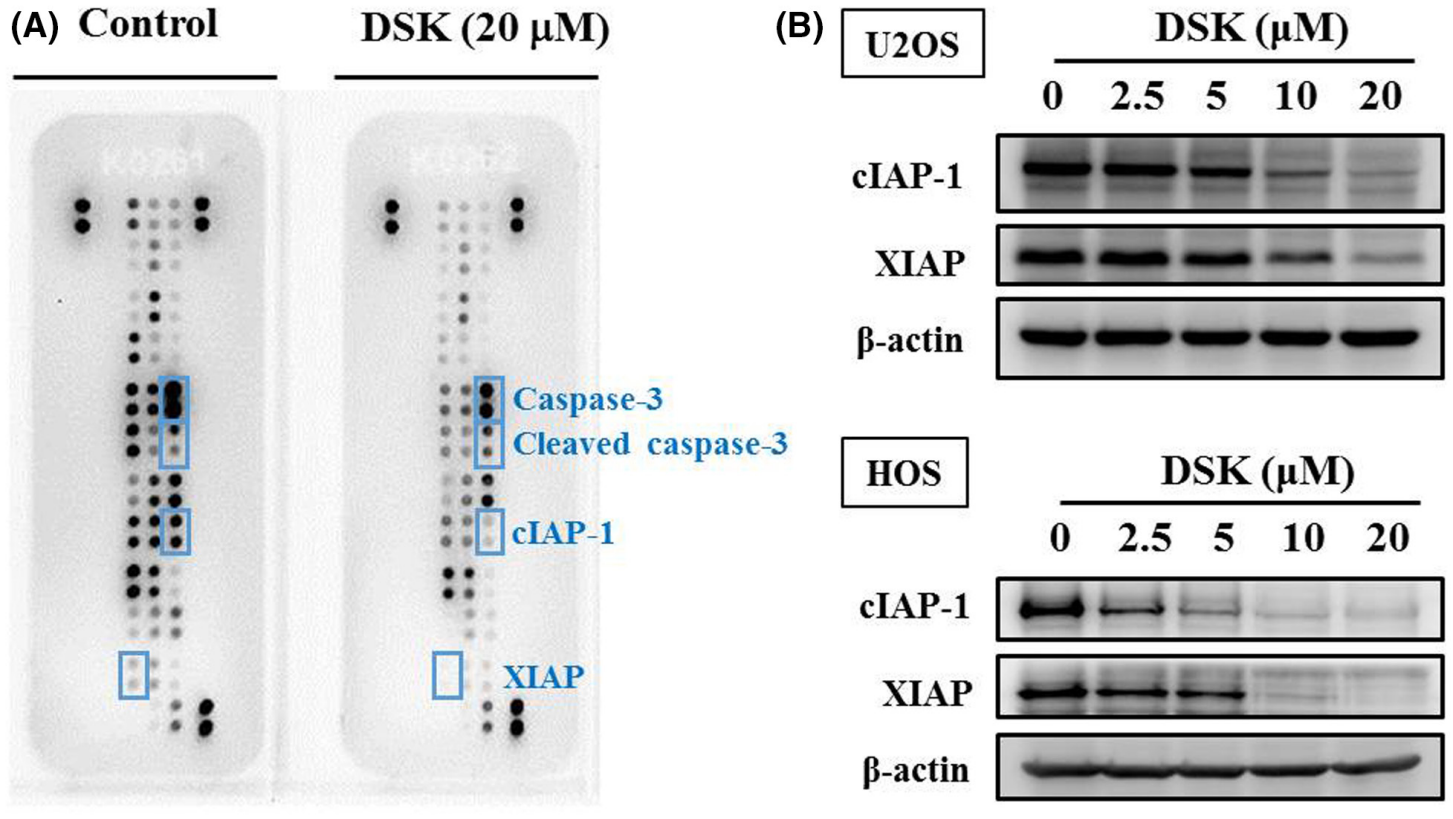
Analysis of deoxyshikonin on the human apoptosis array and inhibitor of apoptosis proteins in HOS cells. (A) After treatment of 20 μM deoxyshikonin for 24 h in HOS cells, the human apoptosis array, 35 apoptosis‐related proteins included, were employed as described in the Section 2 and the increased protein cleaved caspase‐3 and decreased proteins cIAP‐1 and XIAP were subjected to quantitative analysis. (B) Western blot analysis for cIAP‐1 and XIAP after various concentrations (2.5, 5, 10 and 20 μM) of deoxyshikonin treatment for 24 h in U2OS and HOS cells were measured as described in the Section 2.

**FIGURE 5:**
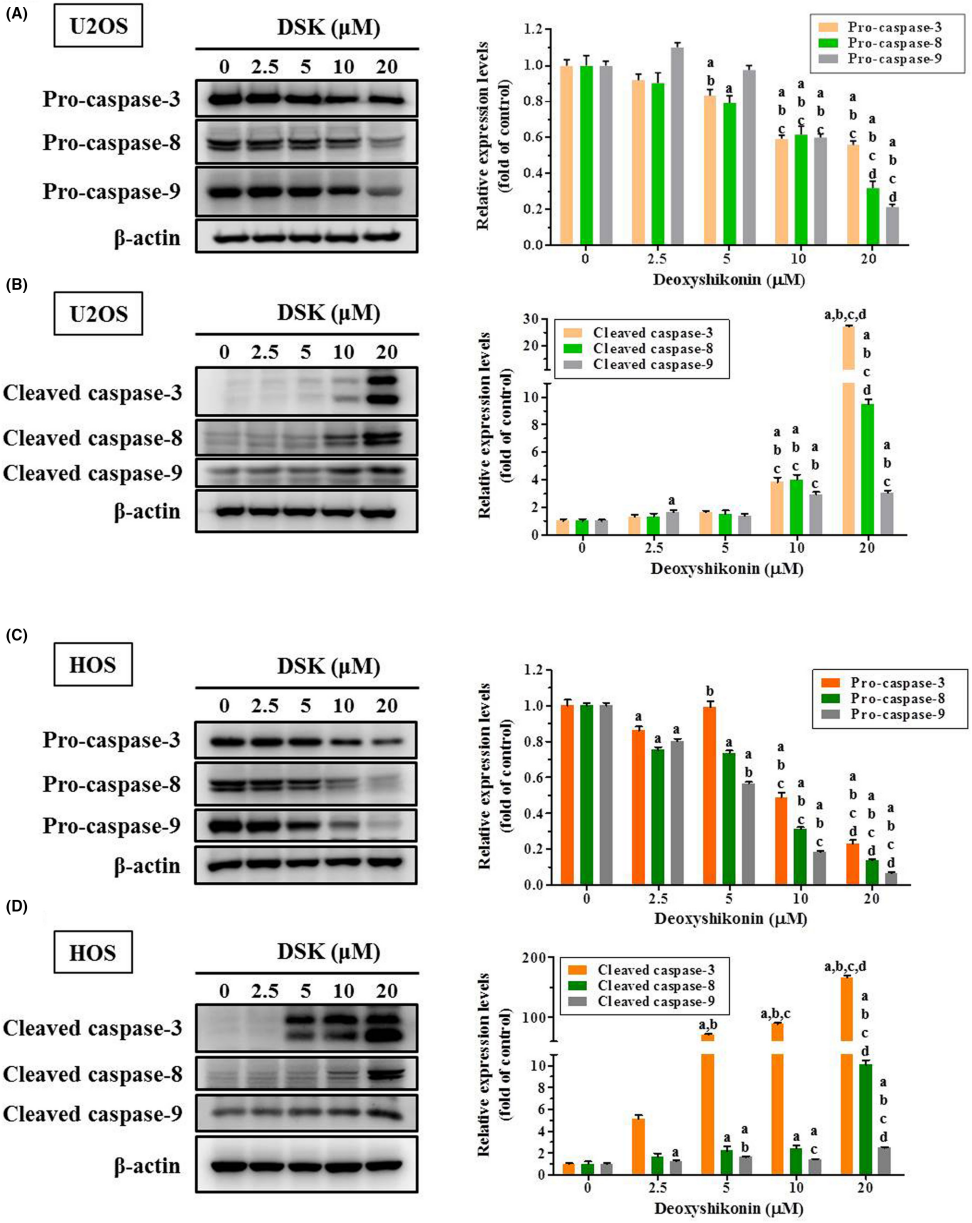
Analysis of deoxyshikonin on activation of caspases 3, 8 and 9 in U2OS and HOS cells. Western blot analysis for (A) caspases 3, 8 and 9 as well as (B) their active forms after various concentrations (2.5, 5, 10 and 20 μM) of deoxyshikonin treatment for 24 h in U2OS and (C, D) HOS cells were measured. All of them were subsequently subjected to quantitative analysis. Results are shown as mean ± SD; *n* = 3. anova analysis with Tukey posteriori comparison was used. U2OS: Caspase 3: *F* = 131.195, *p* < 0.001. caspase 8: *F* = 92.455, *p* < 0.001; caspase 9: *F* = 807.987, *p* < 0.001; Cleaved caspase 3: *F* = 3914.310, *p* < 0.001; cleaved caspase 8: *F* = 445.224, *p* < 0.001; cleaved caspase 9: *F* = 96.686, *p* < 0.001; HOS: Caspase 3: *F* = 433.575, *p* < 0.001. caspase 8: *F* = 1976.101, *p* < 0.001; caspase 9: *F* = 3333.587, *p* < 0.001; Cleaved caspase 3: *F* = 3051.928, *p* < 0.001. cleaved caspase 8: *F* = 347.392, *p* < 0.001; cleaved caspase 9: *F* = 216.919, *p* < 0.001; ^a^Significantly different, *p* < 0.05, when compared to control. ^b^Significantly different, *p* < 0.05, when compared to 2.5 μM. ^c^Significantly different, *p* < 0.05, when compared to 5 μM. ^d^Significantly different, *p* < 0.05, when compared to 10 μM.

To identify the underlying mechanism, we performed the human apoptosis array to examine proteins related to deoxyshikonin apoptosis in HOS cells. Intriguingly, we observed a clear increase in the expression of cleaved caspase 3 in HOS cells after treatment with 20 μM of deoxyshikonin for 24 h, suggesting that deoxyshikonin triggers the caspase cascade in HOS cells (Figure 4A). By Western blotting, dose‐dependent decreases caused by deoxyshikonin in cIAP‐1 and XIAP expressions were verified in U2OS and HOS cells (Figure 4B). We further used Western blot analysis to validate the effect of deoxyshikonin on effector caspase 3 and its upstream initiator caspases 8 and 9 of the apoptotic signalling pathway. After treatment with the experimental concentration range of deoxyshikonin in U2OS and HOS cells for 24 h, deoxyshikonin increased the expressions of the cleaved forms of caspases 3, 8 and 9 in a dose‐dependent manner (U2OS: *p* < 0.001, *p* < 0.001 and *p* < 0.001, respectively; HOS: *p* < 0.001, *p* < 0.001 and *p* < 0.001, respectively), and decreased the expressions of caspases 3, 8 and 9 dose‐dependently (U2OS: *p* < 0.001, *p* < 0.001 and *p* < 0.001, respectively; HOS: *p* < 0.001, *p* < 0.001 and *p* < 0.001, respectively). (Figure 5) Therefore, deoxyshikonin induces apoptosis of U2OS and HOS cells by activating extrinsic caspase 8 and intrinsic caspase 9 mediated cascades and converging on their downstream effector caspase 3.

### 3.4 Deoxyshikonin activates extrinsic and intrinsic apoptotic pathways through p38 signalling in U2OS and HOS cells

**FIGURE 6:**
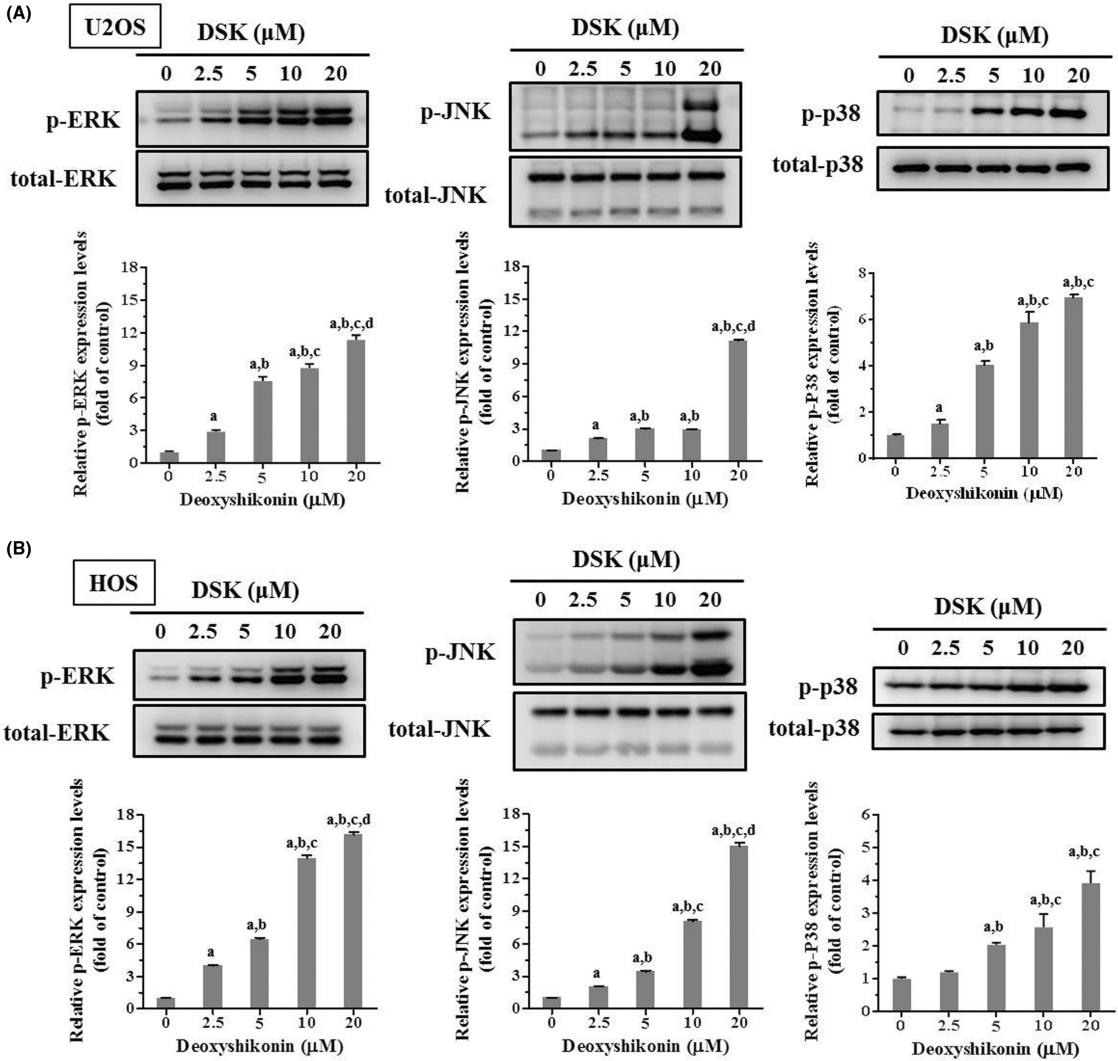
Analysis of deoxyshikonin on the phosphorylation of ERK1/2, JNK1/2 and p38 in U2OS and HOS cells. Expressions of ERK1/2, JNK 1/2 and p38, as well as their phosphorylation after various concentrations (2.5, 5, 10 and 20 μM) of deoxyshikonin treatment for 24 h in (A) U2OS and (B) HOS cells, were measured through Western blot analysis. Next, they were subjected to quantitative analysis. Results are shown as mean ± SD; *n* = 3. anova analysis with Tukey posteriori comparison was used. ^a^Significantly different, *p* < 0.05, when compared to control. ^b^Significantly different, *p* < 0.05, when compared to 2.5 μM. ^c^Significantly different, *p* < 0.05, when compared to 5 μM. ^d^Significantly different, *p* < 0.05, when compared to 10 μM.

MAPKs may form part of the signalling pathways that directly affect caspases 8, 9 and 3 to regulate apoptosis. As shown in Figure 6A,B, we performed Western blot analysis to recognize the role of MAPKs involved in the effect of deoxyshikonin in U2OS and HOS cells. Consequently, deoxyshikonin dose‐dependently increased ERK1/2, JNK1/2 and p38 phosphorylation in U2OS and HOS cells (U2OS: *p* < 0.001, *p* < 0.001 and *p* < 0.001, respectively; *p* < 0.001, *p* < 0.001 and *p* < 0.001, respectively), indicating that MAPK pathways are upregulated by deoxyshikonin in U2OS and HOS cells.

**FIGURE 7:**
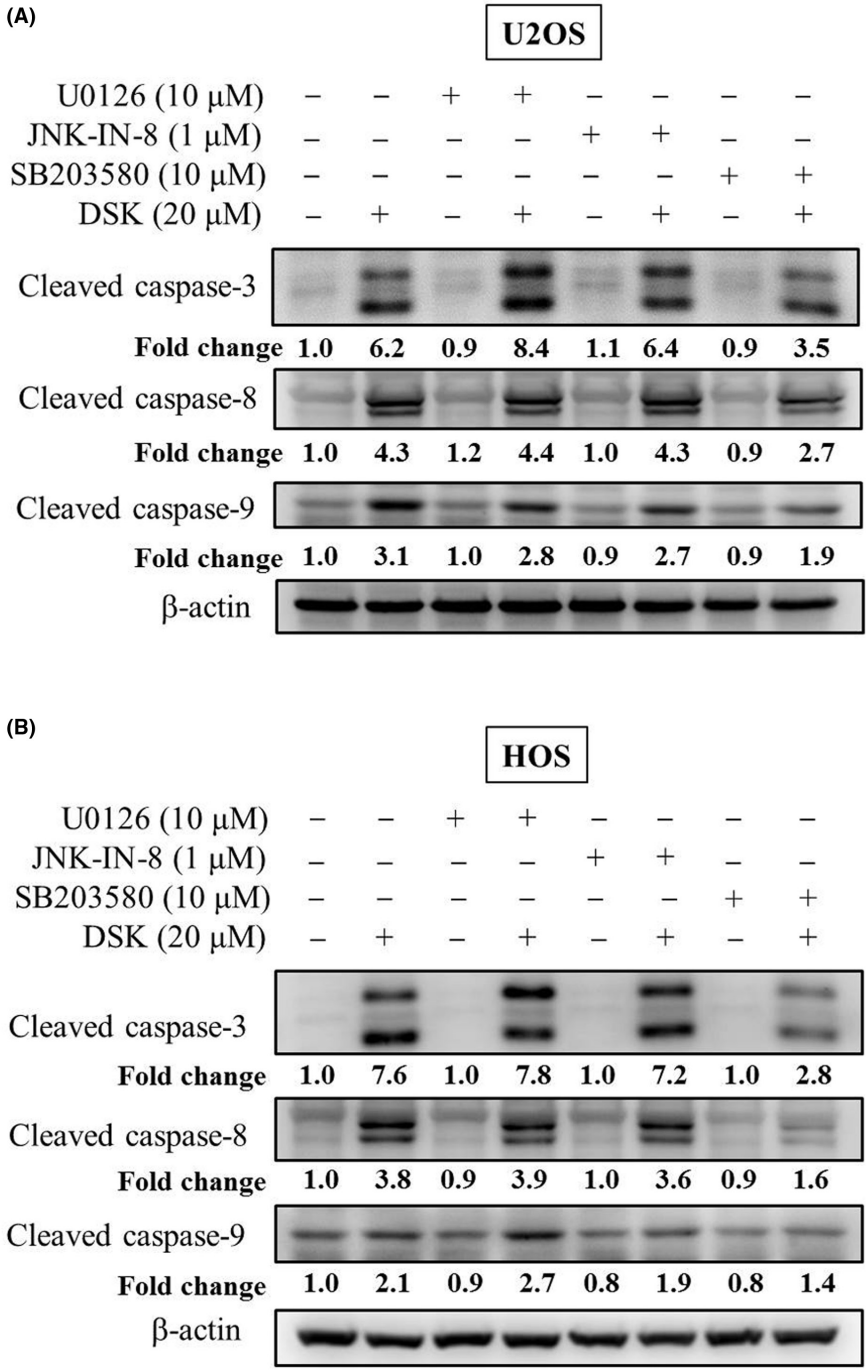
Analysis of deoxyshikonin and inhibitors of ERK1/2 (U0126), JNK1/2 (JNK‐IN‐8) and p38 (SB203580) on cleaved caspases 3, 8 and 9 expressions of (A) U2OS and (B) HOS cells. Expressions of cleaved caspases 3, 8 and 9 after pretreatment with or without 10 μM of U0126, 1 μM of JNK‐IN‐8 and 10 μM of SB203580 for 1 h followed by 20 μM or no deoxyshikonin treatment for an additional 24 h in U2OS and HOS cells were measured through Western blot analysis.

To identify whether increases in phosphorylation of ERK1/2, JNK1/2 and p38 by deoxyshikonin affect the activity of caspases 3, 8 and 9 of extrinsic and intrinsic apoptotic processes in U2OS and HOS cells, we further used inhibitors of ERK1/2 (U0126), JNK1/2 (JNK‐IN‐8) and p38 (SB203580) with or without treatment with 20 μM of deoxyshikonin in Western blot analysis. As expected, cleaved caspases 3, 8 and 9 were observed to be activated by 20 μM of deoxyshikonin as well were repressed by 10 μM of U0126, 1 μM of JNK‐IN‐8 and 10 μM of SB203580 (Figure 7). Intriguingly, 10 μM of SB203580 significantly suppressed the activation of cleaved caspases 3, 8 and 9 caused by 20 μM of deoxyshikonin in U2OS and HOS cells, but 10 μM of U0126 and 1 μM of JNK‐IN‐8 did not suppress the increase of cleaved caspases 3, 8 and 9 in U2OS and HOS cells (Figure 7). Collectively, these results demonstrated that the p38 pathway plays a critical role in deoxyshikonin‐induced apoptosis through extrinsic and intrinsic processes mediated by initiators caspases 8 and 9 and their converging effector caspase 3 in U2OS and HOS cells.

## 4 DISCUSSION

Due to the high number of deaths related to metastasis, patients with powerful metastatic osteosarcoma are eager for new chemotherapeutic remedies to reduce mortality and morbidity. Although medicinal herbs are increasingly used as adjuvant cancer treatments for relative safety, poor solubility in water, low bioavailability and drug resistance reduce the efficacy of additional chemotherapy, such as curcumin and shikonin.^34^, ^35^, ^36^, ^37^, ^38^ Therefore, their various analogues have been developed for cancer cells that increase cytotoxicity, inhibit proliferation, improve sensitized chemotherapy and inhibit metastases.^25^, ^39^, ^40^

In addition to multidrug resistance protein 1 and breast cancer resistance protein (BCRP)1, the new shikonin analog deoxyshikonin bypasses drug resistance through the drug transporters P‐glycoprotein, B‐cell lymphoma (Bcl)‐2 and Bcl‐extra large (Bcl‐xL) to induce necroptosis.^35^ Five shikonin derivatives containing deoxyshikonin exhibit in vitro and in vivo anticancer effects by inducing murine melanoma B16F10 cells apoptosis and arrest in the sub‐G1 phase through repression of Bcl‐2 proteins and activation of Bax expression.^21^ Moreover, through the phosphoinositide 3‐kinase (PI3K)/protein kinase B (Akt)/mammalian target of rapamycin (mTOR) pathway, deoxyshikonin induces apoptosis and cell arrest in the G0/G1 cell cycle in human colorectal cancer HT29 cells and suppresses tumour growth in xenograft mice transplanted with human colorectal cancer DLD‐1 cells.^22^ Deoxyshikonin decreases cell viability and exhibits anti‐glycolytic activity in acute myeloid leukaemia cells by repressing the glycolytic enzyme pyruvate kinase M2 through inactivation of the Akt/mTOR pathway.^23^

In human non‐small cell lung cancer resistant to cisplatin A549/cis and H1299/cis cells with greater resistance to cisplatin, deoxyshikonin contributes to cisplatin‐induced apoptosis and inhibition of cell viability by reducing the expression of the Akt‐mediated adenosine triphosphate (ATP) binding cassette subfamily B member 1.^24^ During apoptosis, cells trigger the initiator and effector caspases by activating the receptor (extrinsic) pathway or stimulating the mitochondria (intrinsic) pathway to undergo cell shrinkage, chromatin condensation, membrane blebbing and nuclear DNA fragmentation.^9^, ^41^, ^42^, ^43^ Signalling of initiator caspases 8 and 9 of both extrinsic and intrinsic pathways converges on their downstream effector caspases 3 or 7.

In the present study, reductions in cell viability, arrest of the cell cycle and induction of apoptosis were observed in cells treated with deoxyshikonin. Apoptosis and cell cycle arrest might be mainly responsible for the deoxyshikonin‐induced inhibition of U2OS and HOS cell growth. Thus, deoxyshikonin could become a new candidate compound against human osteosarcoma and it was necessary to understand the mechanism of anti‐osteosarcoma activity, which induces apoptosis and cell cycle arrest in the sub‐G1 phase by activating both apoptotic pathways in U2OS and HOS cells through p38 signalling.

In conclusion, we demonstrate evidence of the pharmacological effects of deoxyshikonin on human osteosarcoma U2OS and HOS cells by triggering both extrinsic and intrinsic pathways. This phenomenon could be attenuated by the p38 inhibitor but not by the ERK and JNK inhibitors; that is, deoxyshikonin induces apoptosis in U2OS and HOS cells through the p38 pathway. Surely, our results suggest that deoxyshikonin may be a potential anticancer candidate to induce apoptosis of human osteosarcoma. However, the promising anti‐osteosarcoma mechanism of deoxyshikonin needs to be further studied for the successful treatment of human osteosarcoma, and further pharmacological and clinical investigations are required in vivo.

## AUTHOR CONTRIBUTIONS

**Ming‐Chang Hsieh:** Conceptualization (equal); writing – original draft (equal); writing – review and editing (equal). **Yi‐Hsien Hsieh:** Methodology (equal). **Chia‐Hsuan Chou:** Methodology (equal). **Jia‐Sin Yang:** Methodology (equal). **Peace Wun‐Ang Lu:** Methodology (equal). **Tzu‐Yu Huang:** Methodology (equal). **Shun‐Fa Yang:** Conceptualization (equal); writing – original draft (equal); writing – review and editing (equal). **Ko‐Hsiu Lu:** Conceptualization (equal); writing – original draft (equal); writing – review and editing (equal).

## FUNDING INFORMATION

This study was supported by Chung Shan Medical University Hospital, Taiwan (CSH‐2023‐D‐008).

## CONFLICT OF INTEREST STATEMENT

The authors declare that there is no conflict of interest.

## Data Availability

The data used to support the findings of the present study are available from the corresponding author upon request.
